# Effect of replacing peanut vine with extruded rape straw on growth, nutrient digestibility, energy metabolism, microbial crude protein synthesis, meat amino acid and fatty acid profiles of finishing lambs

**DOI:** 10.1093/tas/txaf044

**Published:** 2025-04-14

**Authors:** Daiyi Yang, Tongyu Sun, Yongjie Zheng, Morteza H Ghaffari, Yanling Huang, Yuanfeng Sun, Xiaoyang Li, Tao Ma

**Affiliations:** Key Laboratory of Feed Biotechnology of the Ministry of Agriculture and Rural Affairs, Institute of Feed Research of Chinese Academy of Agricultural Sciences, Beijing 100081, China; Key Laboratory of Feed Biotechnology of the Ministry of Agriculture and Rural Affairs, Institute of Feed Research of Chinese Academy of Agricultural Sciences, Beijing 100081, China; Key Laboratory of Feed Biotechnology of the Ministry of Agriculture and Rural Affairs, Institute of Feed Research of Chinese Academy of Agricultural Sciences, Beijing 100081, China; Institute of Animal Science, Physiology Unit, University of Bonn, Bonn 53115, Germany; College of Animal and Veterinary Sciences, Southwest Minzu University, Chengdu 610041, China; Rural Energy and Environment Agency, Ministry of Agriculture and Rural Affairs, Beijing 100125, China; Rural Energy and Environment Agency, Ministry of Agriculture and Rural Affairs, Beijing 100125, China; Key Laboratory of Feed Biotechnology of the Ministry of Agriculture and Rural Affairs, Institute of Feed Research of Chinese Academy of Agricultural Sciences, Beijing 100081, China

**Keywords:** extruded rape straw, peanut vine, lamb, growth, amino acid, fatty acid

## Abstract

Peanut crop residues such as peanut vine are widely used to feed small ruminants as roughage in Asia, where the population of sheep and goat takes a large proportion in the world. Compared to peanut vine, straw is considered as a less nutritious but promising roughage source due to high availability and low price. Extrusion is a process to improve the palatability and digestibility of the feed. Here, we investigated the effects of replacing peanut vine with extruded rape straw on growth performance, apparent nutrient digestibility, energy metabolism, microbial crude protein (MCP) synthesis, and amino acid and fatty acid profiles in the *longissimus thoracic* (LT) of finishing lambs. Twenty-four 2-mo-old male Hu lambs of similar body weight (19.5 ± 1.0 kg) were fed two diets containing either peanut vine (CON, n = 12) or extruded rape straw (TRT, n = 12) as the only roughage source. The dietary concentrate to forage ratio was 70:30. Lambs were fed twice daily and had ad libitum access to feed and clean water. The experiment lasted 120 d, including a 30-d adaptation period. Lambs were weighed every 30 d. Digestibility trial was conducted during the 81 to 90 d of the experiment. At the end of the experiments, lambs were slaughtered for ruminal fluid and meat sample collection. Data were analyzed using mixed model with repeated measures, Student’s t-test, or Wilcoxon signed-rank test. No difference in growth performance, apparent digestibility of nutrients, or energy utilization efficiency was observed, except for the apparent digestibility of ether extract, which was greater (*P *= 0.008) for lambs in the TRT group. In addition, the MCP estimated using urinary purine derivatives (*P* = 0.072) tended to be greater in the lambs of the TRT group. The concentration of most amino acids and fatty acids was not different in the LT sample of two groups of lambs, while the concentration of Met (*P* = 0.044) was greater, and that of myristoleic acid (C14:1) (*P* = 0.010), heptadecenoic acid (C17:1) (*P* < 0.001), trans-linoleic acid (C18:2n6t) (*P* = 0.003) and gamma-linolenic acid (C18:3n6) (*P* < 0.001) were lower in the LT sample of lambs from TRT group. In conclusion, extruded rape straw can effectively replace peanut vine in the diet of lambs without compromising health or nutrient utilization.

## Teaser

Replacing peanut vine with extruded rape straw did not affect the growth performance of lambs. Replacing peanut vine decreased the contents of multiple unsaturated fatty acids in the *longissimus thoracic* of lambs.

## INTRODUCTION

Agricultural byproducts are increasingly recognized as valuable feed resources for animal production. Optimizing their use can improve the environmental sustainability of ruminant production and reduce costs in intensive livestock farming (Salami et al., 2019). Among these byproducts, rape (*Brassica napus* subsp. *napus*) straw stands out due to its abundance and potential as a feed resource. As the third largest oil crop in the world (Montoya et al., 2021), rapeseed production generates about 210 million tons of rape straw annually (Huang et al., 2021). China, as the leading producer of rapeseed, contributes around 21 million tons to this amount (Wang et al., 2020). However, less than 25% of this straw is currently utilized, with the majority being incinerated, resulting in economic losses and significant greenhouse gas emissions (Huang et al., 2021).

Ruminants have a specialized microbiota that enables them to efficiently break down lignocellulose, a complex carbohydrate in straw that is not digestible by humans and monogastric animals (Matthews et al., 2019). This ability makes straw-based roughage a promising feed source for ruminants. Rape straw, like other crop straws, is rich in dietary fiber with neutral detergent fiber (NDF) and acid detergent fiber (ADF) contents of up to 82% and 65%, respectively (Lan et al., 2019). However, the presence of anti-nutritional factors such as glucosinolates and sinapine may limit the voluntary intake of rape straw by ruminants. Despite these challenges, there is little research on effective processing methods that could improve the nutritional value and utilization of rape straw in ruminant diets.

To overcome these challenges, extrusion has been identified as a pretreatment method that can break down the lignocellulosic structure, resulting in defibration and shortening of the fibers in the straws (Hjorth et al., 2011; Chen et al., 2014). This process has been shown to improve milk production, lactose concentration in milk and digestibility of dry matter (DM) and crude protein (CP) in dairy cows (Risyahadi et al., 2023). However, most studies have focused on the extrusion of concentrates such as corn, soybean meal or wheat (Risyahadi et al., 2023).

Peanut vine, a roughage source widely used in sheep and goat production in Asia (Khan et al., 2013; Zhu et al., 2022) where the population of sheep (~42%) and goats (~60%) account for a large proportion of the total population in the world (FAOSTAT, 2023). On the contrary, high-quality roughage such as alfalfa and barley are rarely used in sheep and goat production in these regions due to high cost. Our previous research has shown that extrusion can reduce ADF and glucosinolate content in rape straw (Yang et al., 2024). Building on this, we investigated the effects of replacing peanut vine with extruded rape straw in the diet of finishing lambs. We hypothesized that lambs fed extruded rape straw would have comparable growth performance and nutrient utilization to lambs fed peanut vine, with potential changes in amino acid and fatty acid profiles due to dietary differences. The objective of this study was to evaluate the potential of extruded rape straw as an alternative to peanut vine in the diet of lambs by investigating the effects on growth performance, nutrient digestibility, energy metabolism, and muscle amino acid and fatty acid profiles.

## MATERIALS AND METHODS

### Ethics Statement

This experiment was conducted according to the ARRIVE (Animal Research Reporting of in Vivo Experiments) guidelines from June to October 2023 at the experimental station of the Institute of Feed Research of CAAS in Beijing (N40.23, E116.10). The protocols for feeding and slaughter were approved by the Animal Ethics Committee of the Institute of Feed Research of the CAAS (protocol number: IFR-CAAS-20230611).

### Animals, Experimental Design, and Diets

Twenty-four 2-mo-old male Hu lambs of similar body weight (19.5 ± 1.0 kg) were used in this study and fed two diets containing either peanut vine (CON, n = 12) or extruded rape straw (TRT, n = 12) as the sole source of roughage. The rape straw was extruded according to the following procedure: 30% of water was added per tonne of rape straw, which was extruded under 150 to 200 °C and 15 Mpa. Approximately 800 kg of extruded rape straw could be produced per hour (Model 9P-150, Kunzhentianxi, Inner Mongolia, China). The experimental diets were formulated to achieve an average daily gain (ADG) of 200 g/d for the male fattening lambs according to the national feeding standard (Ma et al., 2022). Dietary concentrate to roughage ratio was 70:30. The ingredients and nutrient contents of the experimental diets as total mixed ratio (TMR), as the nutrient contents of different roughage sources are shown in Table 1. Each lamb was kept in a separate pen (2.5 m × 1.0 m) and served as a replicate. All lambs were fed twice daily (8:00 am and 6:00 pm) and had ad libitum access to diet (adjusted to less than 10% of refusals daily) and clean water.

**Table 1.** Ingredients of experimental diets and nutritional level of diets and roughages (DM basis)

Item	Groups^1^		Peanut vine	Rape straw	Extruded rape straw
	CON	TRT			
Ingredients					
Peanut vine	30.0	–			
Extruded rape straw	–	30.0			
Corn	41.0	39.5			
Soybean meal	15.0	16.5			
Wheat bran	10.0	10.0			
Premix^2^	4.0	4.0			
Total	100.0	100.0			
Nutritional level					
ME, MJ/kg of DM^3^	11.2	10.7	–	–	–
DM, %	86.2	86.3	92.7	91.9	92.6
Ash, %	4.95	4.51	8.76	7.09	7.31
CP, %	12.9	13.0	7.62	6.99	5.29
EE, %	2.6	2.7	2.57	2.69	2.52
NDF, %	27.9	30.7	50.8	74.3	71.5
ADF, %	17.6	20.1	39.4	57.8	60.3
Ca, %	1.1	1.1	0.74	0.80	0.82
P, %	0.5	0.5	0.10	0.11	0.11

ME = metabolizable energy; DM = dry matter; CP = crude protein; EE = ether extract; NDF = neutral detergent fiber; ADF = acid detergent fiber.

^1^CON, peanut vine as the only roughage source; TRT, extruded rape straw as the only roughage source.

^2^The premix provided the following per kg of diets: VA 15 000 IU, VD 2200IU, VE 50 IU, Fe 55 mg, Cu 12.5 mg, Mn 47 mg, Zn 24 mg, Se 0.5 mg, Co 0.1 mg.

^3^ME (MJ/kg) was calculated as ME intake (MJ/d)/DMI (kg/d) during the digestibility trial.

### Experimental Procedure and Sample Collection

The experiment lasted 120 d, including a 30-d adaptation period. After the adaptation period, the amount of feed offered and refused was recorded daily for each lamb. The lambs were weighed at the start as well as after 30, 60, and 90 d of the experiment to calculate ADG and feed conversion ratio (FCR).

During the 81 to 90 d of the experiment, 5 lambs from each group that with similar weight were randomly selected and placed in the metabolic crates (1.45 m × 1.25 m × 0.6 m, height × length × width) equipped with separate compartments for collecting feces and urine. After an adaptation period of 4 d, the offered and refused feed and feces were weighed daily, homogenized and 10% of these samples were collected during a 6-d collection period. Similarly, urine was collected daily in buckets containing 100 mL of 10% (v/v) H_2_SO_4_ for 6 consecutive days. The urine volume was measured and a subsample (10 mL/L of the total volume) was collected. Feed, ort, feces, and urine samples from each lamb were pooled and stored at −20 °C until analysis.

At the end of the experiment, 10 lambs from each group were randomly selected, fasted for 12 h, and had free access to water before slaughtered. After exsanguination, the rumen was removed and opened, and two 10-mL tubes of rumen fluid were collected by squeezing the ruminal digest from the dorsal sac through four layers of cheesecloth. The pH value in the rumen was measured immediately using one of the tubes with the collected samples. In addition, approximately 200 g of *longissimus thoracic* (LT) meat was sampled between the 11th and 12th ribs on the right side of each lamb carcass. The other tube containing the ruminal fluid and meat samples was stored at −20 °C for further analysis.

### Measurements

The gross energy (GE) content of the feed, orts, urine, and fecal samples was measured using a bomb calorimeter (C200, IKA Works Inc., Staufen, Germany). The GE in solid samples were determined by directly burning the sample and measuring the heat of combustion. To measure the GE in urine samples, a cellulose pellet with or without drips of urine were ignited in a bomb calorimeter and the difference of calorific values was calculated. The DM content of the feed, orts, and fecal samples was determined by drying at 135 °C for 2 h according to method 930.15 of AOAC 1990. Nitrogen content in the feed, orts, urine, and fecal samples was determined using the Kjeldahl method (Method 991.20; AOAC 1990), and CP was calculated as 6.25 times the nitrogen content. The ether extract (EE) content was defined as the DM weight loss after extraction with diethyl ether in a Soxhlet extraction apparatus for 8 h (method 920.85; AOAC, 1990). The ash content of the diet was determined by incinerating the samples at 550 °C for 4 h (Method 942.05; AOAC 1990), and the organic matter (OM) content was calculated as 100% minus the ash percentage. The NDF and ADF content of the feed, orts, and fecal samples were determined according to the methods of Van Soest et al. (1991) and Goering and Van Soest (1970), respectively. Urinary excretion of purine derivatives (PD) including allantoin, uric acid, xanthine, and hypoxanthine was quantified separately by the colorimetric method as described by Chen and Gomes (1995). Briefly, allantoin was degraded to urea and glyoxylic acid which then reacted with phenylhydrazine hydrochloride to produce a phenylhydrazone of the acid. The product formed an unstable chromophore with potassium ferricyanide and the color was read at 522 nm. To measure xanthine and hypoxanthine, urinary samples were treated with xanthine oxidase, xanthine and hypoxanthine were then converted to uric acid, which was monitored by its absorbance at 293 nm. To measure uric acid, urinary samples were treated with uricase, and uric acid was degraded to allantoin and other compounds that do not absorb UV at 293 nm. Therefore, the reduction in OD reading after treatment with uricase was related with the concentration of uric acid in the sample.

To analyze the amino acid content in the diet and LT meat samples, 150 mg of the oven-dried dietary sample or lyophilized meat powder sample was dissolved in 10 mL of 6 N HCl and then hydrolyzed under nitrogen at 110 °C for 24 h. The pH was then adjusted to 2.2 with 7.5 N NaOH. 0.5 mL of the internal standard (Norleucine 50 M) was added and the samples were diluted with citrate buffer. Finally, the solution was filtered. A 20 L aliquot of the filtrate was analyzed using an amino acid analyzer (L-8900, Shimadzu, Tokyo, Japan) equipped with a sodium-oxidized column, a cation exchange resin, and post-column derivatization of the amino acids to ninhydrin and spectrophotometric detection at 570 nm, except for proline, which was detected at 440 nm. The amino acid profiles of the experimental diets are shown in Table 2.

**Table 2.** Amino acid profiles of experimental diets (g/100 g of protein)

Item	Groups^1^	
	CON	TRT
Asp	1.70	1.47
Thr	0.67	0.61
Ser	0.99	0.70
Glu	2.91	3.11
Gly	0.86	0.40
Ala	1.30	0.86
Val	1.13	0.73
Met	0.46	0.29
Ile	1.43	0.78
Leu	2.15	1.06
Tyr	0.87	0.32
Phe	1.27	0.91
Lys	0.94	0.81
His	0.88	0.31
Arg	1.55	0.99
Pro	1.84	1.80
Total	20.90	15.12

^1^CON, peanut vine as the only roughage source; TRT, extruded rape straw as the only roughage source.

The fatty acid contents in the diet and LT meat samples was determined as described by Folch et al (1957). The fatty acid profile of the procured samples was analyzed using a gas chromatograph (GC-2030, Shimadzu, Tokyo, Japan) equipped with a flame ionization detector. A homogeneous sample of 2.5 g of LT meat was weighed and added to a 250-mL flat-bottomed flask together with 1.0 mL internal standard solution of triglycerol undecanoate. Subsequently, 100 mg pyrogallic gallic acid, 2 mL 95% ethanol, 4 mL water and 10 mL hydrochloric acid solution were added to the flask, which was then hydrolyzed in a water bath at 70 °C to 80 °C for 40 min. The flask was shaken every 10 min to prevent the particles from adhering to the wall. After hydrolysis, the flask was cooled to room temperature and 8 mL of a 2% sodium hydroxide-methanol solution was added to the fat extract, after which the reflux condenser was connected. The mixture was refluxed on a water bath at 80 °C ± 1 °C until the oil droplets disappeared, and then 7 mL of a 15% boron trifluoride-methanol solution was added to the top of the reflux condenser. The mixture was then refluxed for a further 2 min at 80 °C ± 1 °C, rinsing the reflux condenser with a small amount of water. The flask was removed from the water bath and rapidly cooled to room temperature before adding 10 to 30 mL of n-heptane and shaking the flask for 2 min. Saturated aqueous sodium chloride solution was then added and the mixture was allowed to stratify. The upper layer of the n-heptane extraction solution (about 5 mL) was pipetted into a test tube. About 3 to 5 g of anhydrous sodium sulfate was added to the test tube and shaken for 1 min. The mixture was allowed to stand for 5 min and then the upper layer of the solution was pipetted into the injection vial to measure the fatty acid profiles. The fatty acid profiles of the experimental diets are shown in Table 3.

**Table 3.** Fatty acid profiles of experimental diets (g/100 g of fat)

Item^2^	Groups^1^	
	CON	TRT
C4:0	ND	ND
C6:0	ND	ND
C8:0	ND	ND
C10:0	ND	ND
C11:0	ND	ND
C12:0	ND	ND
C13:0	ND	ND
C14:0	0.0049	0.0023
C15:0	0.0029	0.0015
C16:0	0.432	0.184
C17:0	ND	ND
C18:0	0.0708	0.0286
C20:0	0.0122	0.0070
C21:0	0.0020	0.0074
C22:0	ND	0.0036
C23:0	ND	ND
C24:0	ND	0.0118
C14:1	ND	ND
C15:1	ND	ND
C16:1	0.0031	0.0023
C17:1	ND	ND
C18:1n9t	0.0016	ND
C18:1n9c	0.559	0.224
C18:2n6t	ND	ND
C18:2n6c	ND	0.317
C18:3n6	ND	ND
C18:3n3	0.0384	0.0190
C20:1	0.0080	0.0053
C20:2	ND	ND
C20:3n6	ND	ND
C20:3n3	ND	ND
C20:4n6	ND	ND
C20:5n3	ND	ND
C22:1n9	0.0016	0.0062
C22:2	ND	ND
C22:6n3	ND	ND
C24:1	0.0058	0.0028
SFA^2^	0.5248	0.2462
MUFA^3^	0.5791	0.2406
PUFA^4^	0.0384	0.3360
TFA^5^	1.142	0.822
SFA/TFA	0.459	0.299
(MUFA + PUFA)/TFA	0.541	0.701

^1^ND = not detected; CON, peanut vine as the only roughage source; TRT, extruded rape straw as the only roughage source; TFA, total fatty acids, SFA, saturated fatty acids, MUFA, monounsaturated fatty acids; PUFA, polyunsaturated fatty acids.

^2^SFA is the sum of C4:0, C8:0, C10:0, C11:0, C12:0, C13:0, C14:0, C15:0, C16:0, C17:0, C18:0, C21:0, C22:0, C23:0, and C24:0.

^3^MUFA is the sum of C14:1, C15:1, C16:1, C17:1, C18:1n9t, C18:1n9c, C20:1, C22:1n9, and C24:1.

^4^PUFA is the sum of C18:2n6t, C18:2n6c, C18:3n6, C18:3n3, C20:2, C20:3n6, C20:3n3, C20:4n6, C20:5n3, C22:2, and C22:6n3.

^5^TFA is the sum of SFA, MUFA, and PUFA.

### Data Calculation and Statistical Analysis

The digestible energy (DE) intake scaled to MJ/(kgW^0.75^·d) was calculated as the difference between GE intake and fecal energy (FE) output. Metabolizable energy (ME) intake was calculated as the difference between DE intake and urinary energy (UE) and methane energy (CH_4_-E) output. CH_4_ production was calculated using the prediction equation established by Ma et al. (2019):


$$\begin{array}{lll} CH_{4}\left(L/d\right) & \;=\;--6.20\;+\;0.027\;\times \;DM\;intake\;\left(DMI,\;g/d\right)\; & \;+\;0.039\;\times \;NDF\;intake\;\left(NDFI,\;g/d\right). \end{array}$$

The CH_4_-E was then calculated as:


<tex−math>

The MCP synthesis was assessed using urinary excretion of PD. The absorbed microbial PD (X, mmol/d), related to the excretion of PD in urine (Y, mmol/d), was calculated using the equation:


$$\begin{array}{l} Y\;=\;0.84X\;+\;\left(0.150\;\times \;BW^{0.75}\;\times \;e^{\text{\textasciitilde{}}\text{-}0.25x}\right) \end{array}$$

The microbial nitrogen (MN, g/d) was then calculated based on the equation proposed by Chen et al (1992):


$$\begin{array}{l} MN\;=\;\left(70\;\times \;X\right)/\left(0.116\;\times \;0.83\;\times \;1000\right)\;=\;0.727X, \end{array}$$

where the digestibility of microbial purines is assumed to be 0.83 and the N content of purines is 70 mg N/mmol. The MCP was then calculated as MN × 6.25 and scaled to metabolic BW.

Data including BW, DMI, ADG, and FCR were analyzed using mixed model with repeated measures in R Studio using “lmer” function followed by the TukeyHSD post-hoc test using ‘lsmeans’ function in R studio. For the other data, the Shapiro-Wilk test was initially employed to assess the normality of residuals. Among all variables, the residuals of the concentration of amino acids including Gly, Thr, Phe, His, Arg, Pro and the ratio of essential amino acids (EAA) to non-essential amino acids (NEAA), and the concentrations of fatty acids including C15:0, C17:0, C24:0, C14:1, C17:1, C18:1n9t, C18:2n6t, C18:2n6c, C18:3n6, C20:3n6, C20:4n6, and polyunsaturated fatty acids (PUFA) were not normally distributed, while the residuals of other data were normally distributed. For normally distributed datasets, a Student’s t-test was applied. For non-normally distributed datasets, the Wilcoxon signed-rank test was used. Statistical significance was determined at *P* < 0.05 and trends were declared at 0.05 < *P* ≤ 0.10.

The differences in total amino acids (TAA), EAA, NEAA, the EAA/NEAA ratio, total fatty acids (TFA), saturated fatty acids (SFA), monounsaturated fatty acids (MUFA), and PUFA in the LT muscle of lambs between treatment groups were tested using the Mann-Whitney U test. The effect size for these differences was calculated using Hedges’ g (Fritz et al., 2012; Claridge-Chang and Assam, 2016) to quantify the magnitude of differences between treatment groups (TRT vs. CON) and categorized as “trivial” (|*g*| < 0.2), “small” (0.2 < |*g*| < 0.5), “moderate” (0.5 < |*g*| < 0.8), or “large” (|*g*| > 0.8). Additionally, 95% confidence intervals for mean differences were determined using bootstrap methods (5,000 resamples, bias-corrected, and accelerated).

## RESULTS

### Growth Performance of Lambs

**Figure 1.**
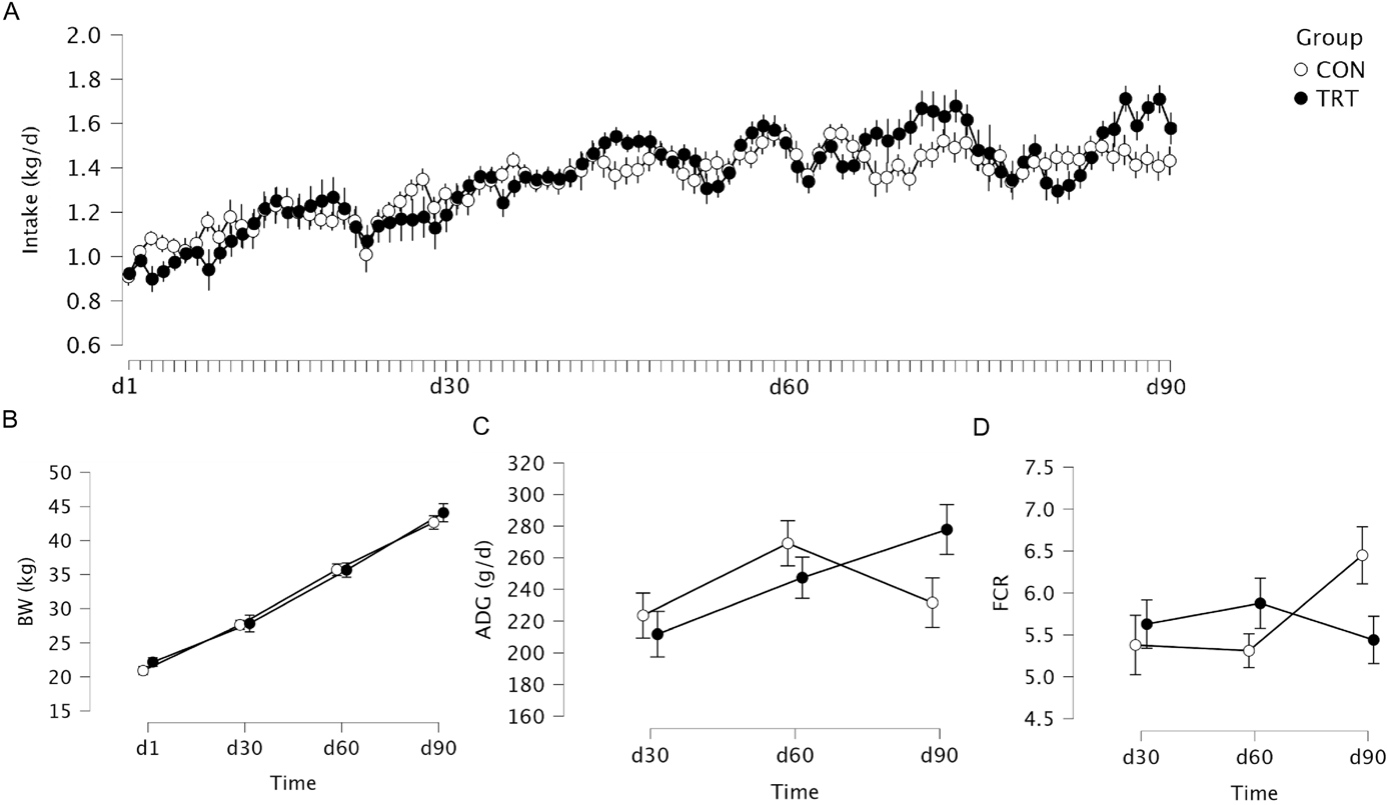
Intake, body weight (BW), average daily gain (ADG), and feed conversion ratio (FCR) of lambs fed different sources of roughages. CON, peanut vine as the only roughage source; TRT, extruded rape straw as the only roughage source.

Both roughage source and time interactively affected average DMI (*P* < 0.001), OMI (*P* < 0.001), NDFI (*P* < 0.001), EEI (*P* < 0.001), ADG (*P* = 0.012), and FCR (*P* = 0.029), but not BW (*P* = 0.326) (Table 4). Specifically, the ADG was greater at d90 than d30 for lambs in the TRT group (*P* = 0.004), but not in the CON group (*P* = 0.996). In addition, the FCR tended to be greater at d60 (*P* = 0.066) and d90 (*P* = 0.093) than at d30 in the CON group, while such a pattern was not observed in the TRT group. Differences in BW, DMI, ADG, and FCR at each time point are shown in Figure 1.

**Table 4.** Effects of replacing peanut vine by extruded rape straw on growth performance of lambs

Items	Groups^1^		SEM	*P*-value		
	CON	TRT		Group	Time	Group × Time
IBW, kg	20.9	22.2	0.41	0.449	–	–
FBW, kg	42.7	44.1	0.73	0.163	–	–
DMI, g/d	1338	1355	5.66	0.089	<0.001	<0.001
OMI, g/d	1271^b^	1294^a^	5.39	0.021	<0.001	<0.001
NDFI, g/d	373^b^	416^a^	1.73	<0.001	<0.001	<0.001
EEI, g/d	34.8^b^	36.6^a^	0.15	<0.001	<0.001	<0.001
ADG, g/d	241.5	244.6	0.18	0.790	0.002	0.012
FCR, kg DMI/kg BW gain	5.55	5.54	0.10	0.812	0.488	0.031

IBW = initial bodyweight; FBW = final bodyweight; DMI = dry matter intake; OMI = organic matter intake; NDFI = neutral detergent fiber intake; EEI = ether extract intake; ADG = average daily gain; FCR = feed conversion ratio.

^1^CON, peanut vine as the only roughage source; TRT, extruded rape straw as the only roughage source.

^a,b^Values in the same row with no common superscripts mean significant difference (*P* < 0.05).

### Apparent Nutrient Digestibility

No difference in the apparent digestibility of DM (*P* = 0.602), OM (*P* = 0.482), NDF (*P* = 0.453), or ADF (*P* = 0.769) was observed between the two groups of lambs (Table 5). The apparent digestibility of EE was greater (*P *= 0.008) and that of CP tended to be greater (*P* = 0.066) for lambs in the TRT than those in the CON group.

**Table 5.** Effects of replacing peanut vine by extruded rape straw on apparent digestibility of lambs

Items	Groups^1^		SEM	*P*-value
	CON	TRT		
DM, %	78.8	78.1	0.63	0.602
OM, %	80.5	79.9	0.53	0.482
EE, %	77.8^b^	80.7^a^	0.72	0.002
CP, %	69.9	73.8	1.01	0.084
NDF, %	52.6	50.7	1.26	0.453
ADF, %	56.2	55.6	0.80	0.769

DM = dry matter; OM = organic matter; CP = crude protein; EE = ether extract; NDF = neutral detergent fiber; ADF = acid detergent fiber.

^1^CON, peanut vine as the only roughage source; TRT, extruded rape straw as the only roughage source.

^a,b^Values in the same row with no common superscripts mean significant difference (*P* < 0.05).

### Energy Metabolism

The lambs in the TRT group had significantly greater GE intake (*P* = 0.037), fecal energy (FE) output (*P* = 0.032), methane energy (CH_4_-E) output (*P* = 0.023), and greater DE intake (*P* = 0.044) and the ratio of CH_4_-E to GE intake (*P* = 0.003) (Table 6). The ME intake of lambs in the TRT group tended to be greater than those in the CON group (*P* = 0.057). There were no significant differences in the UE output (*P* = 0.115), the ratio of DE intake to GE intake (*P *= 0.209), the ratio of ME intake to GE intake (*P* = 0.191), or the ratio of ME intake to DE intake (*P* = 0.397) between two groups of lambs.

**Table 6.** Effects of replacing peanut vine by extruded rape straw on energy metabolism of lambs

Items	Groups^1^		SEM	*P*-value
	CON	TRT		
GE intake, MJ/(kgBW^0.75^·d)	1.239^b^	1.529^a^	0.073	0.037
FE output, MJ/(kgBW^0.75^·d)	0.258^b^	0.339^a^	0.020	0.032
UE output, MJ/(kgBW^0.75^·d)	0.089	0.108	0.006	0.115
CH_4_-E output, MJ/(kgBW^0.75^·d)^2^	0.089^b^	0.116^a^	0.006	0.023
DE intake, MJ/(kgBW^0.75^·d)	0.981^b^	1.190^a^	0.054	0.044
ME intake, MJ/(kgBW^0.75^·d)	0.803	0.965	0.044	0.057
CH_4_-E output/GE intake, %	7.13^b^	7.57^a^	0.085	0.003
DE intake/GE intake, %	79.3	77.8	0.550	0.209
ME intake/GE intake, %	65.0	63.1	0.681	0.191
ME intake/DE intake, %	81.9	81.1	0.455	0.397

BW = bodyweight; GE = gross energy; FE = fecal energy; UE = urinary energy; CH_4_-E = methane energy; DE = digestible energy; ME = metabolizable energy.

^1^CON, peanut vine as the only roughage source; TRT, extruded rape straw as the only roughage source.

^2^CH_4_ output was calculated according to Ma et al. (2019): CH_4_ (L/d) = −6.2 + 0.027 × DMI (kg/d) × 1000 (kg/d) + NDF intake (kg/d) × 0.039 × 1000. The CH_4_-E output was calculated as: CH_4_-E (MJ/d) = [CH_4_ (L/d) × 0.668 (kg/m^3^)]/1000 × 55.65 (MJ/kg).

^a,b^Values in the same row with no common superscripts mean significant difference (*P* < 0.05).

*3.4. Effects on urinary excretion of purine derivatives and microbial protein synthesis* Urinary excretion of allantoin (*P* = 0.032) and total PD (*P* = 0.078) was greater and tended to be greater in lambs in the TRT group than in the CON group, respectively (Table 7). No difference was observed in urinary excretion of uric acid (*P* = 0.285) or xanthine + hypothanxine (*P* = 0.377). The proportion of allantoin was greater (*P* = 0.025), while that of uric acid (*P* = 0.014) and xanthine + hypothanxine (*P* = 0.036) was lower in the lambs of the TRT group than in those of the CON group. The MCP tended to be greater in the lambs in the TRT group (*P* = 0.072).

**Table 7.** Effects of replacing peanut vine by extruded rape straw on urinary excretion of purine derivatives and microbial crude protein synthesis of lambs

Items	Groups		SEM	*P*-value
	CON	TRT		
Excretion, mmol/d				
Allantoin	8.81	12.1	0.95	0.032
Uric acid	1.87	1.24	0.23	0.285
Xanthine + hypoxanthine	0.137	0.145	0.018	0.377
Total PD	10.8	13.5	1.32	0.078
Proportion of each PD, %				
Allantoin	81.6^b^	89.6^a^	2.33	0.025
Uric acid	17.3^a^	9.18^b^	2.57	0.014
Xanthine + hypoxanthine	1.27^a^	1.07^b^	0.074	0.036
MCP synthesis, g/d^2^	42.7	49.2	1.39	0.072

PD = purine derivative; MCP = microbial protein synthesis.

^1^CON, peanut vine as the only roughage source; TRT, extruded rape straw as the only roughage source.

^2^The amount of absorbed microbial PD (X, mmol/d) related to excretion of PD in urine (Y, mmol/d) was calculated as: Y = 0.84X + (0.150BW^0.75^e^−0.25x^). The microbial nitrogen (MN, g/d) was then calculated as: MN = (70 × X)/(0.116 × 0.83 × 1000) = 0.727X. The MCP was finally calculated as MN × 6.25 and scaled to metabolic BW.

^a,b^Values in the same row with no common superscripts mean significant difference (*P* < 0.05).

### Amino Acid Profiles in Longissimus Thoracic

**Figure 2.**
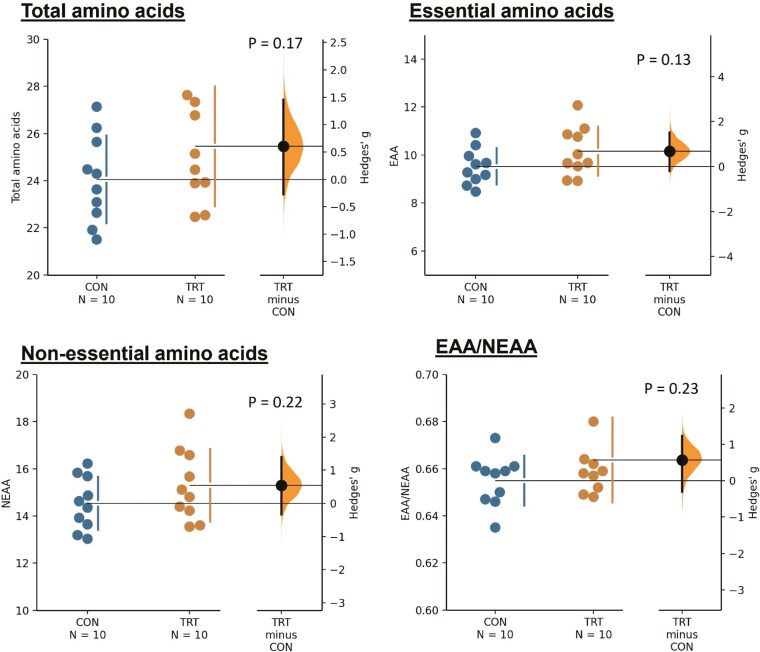
Concentrations of total amino acids (TAA), essential amino acids (EAA), non-essential amino acids (NEAA), and the EAA/NEAA ratio in the *longissimus thoracic* muscle of lambs fed different roughage sources: peanut vine (CON) and extruded rape straw (TRT). Hedges’ *g* (effect size) for 2 comparisons (TRT vs. CON) is shown in the Cumming estimation plot. The raw data are plotted on the upper axes; each Hedges’ *g* is plotted on the lower axes as a bootstrap sampling distribution. Mean differences are depicted as dots; 95% CI are indicated. The unit of total and individual amino acid is expressed as g/100 g fresh sample. EAA is the sum of Val, Met, Ile, Leu, Phe, Lys, and Thr. NEAA is the sum of Asp, Ser, Glu, Pro, Gly, Ala, Tyr, His, and Arg.

The concentration of Met (*P* = 0.044) was greater, while that of Tyr tended to be greater (*P* = 0.088) in the LT samples, and no difference was observed in that of TAA (*P* = 0.17), EAA (*P* = 0.13), NEAA (*P* = 0.22), EAA/NEAA (*P* = 0.23) and any other amino acids (Figure 2; Table 8).

**Table 8.** Effects of replacing peanut vine by extruded rape straw on amino acid profiles (g/100 g of protein) in the *longissimus thoracic* of lambs

Items^2^	Groups^1^		SEM	*P*-value
	CON	TRT		
Asp	2.17	2.31	0.024	0.131
Thr	1.20	1.26	0.024	0.181
Ser	1.19	1.26	0.085	0.153
Glu	4.24	4.41	0.022	0.318
Gly	1.18	1.26	0.026	0.151
Ala	1.37	1.44	0.025	0.199
Val	1.17	1.25	0.028	0.108
Met	0.49^b^	0.60^a^	0.026	0.044
Ile	1.29	1.37	0.036	0.106
Leu	1.88	1.97	0.022	0.260
Tyr	0.86	0.94	0.062	0.088
Phe	1.10	1.26	0.056	0.257
Lys	2.40	2.44	0.028	0.723
His	0.84	0.84	0.067	0.520
Arg	1.52	1.60	0.025	0.734
Pro	1.17	1.23	0.510	0.325

^1^CON, peanut vine as the only roughage source; TRT, extruded rape straw as the only roughage source.

^a,b^Values in the same row with no common superscripts mean significant difference (*P* < 0.05).

### Fatty Acid Profiles in Longissimus Thoracic

**Figure 3.**
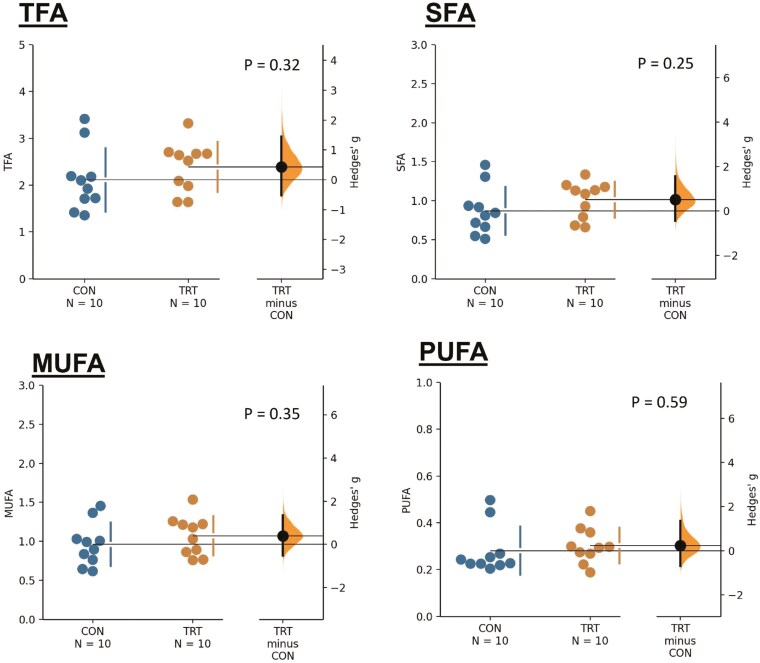
Concentrations of total fatty acids (TFA), saturated fatty acids (SFA), monounsaturated fatty acids (MUFA), and polyunsaturated fatty acids (PUFA) in the *longissimus thoracic* muscle of lambs fed different roughage sources: peanut vine (CON) and extruded rape straw (TRT). Hedges’ *g* (effect size) for 2 comparisons (TRT vs. CON) is shown in the Cumming estimation plot. The raw data are plotted on the upper axes; each Hedges’ *g* is plotted on the lower axes as a bootstrap sampling distribution. Mean differences are depicted as dots; 95% CI are indicated. The unit of total and individual fatty acid is expressed as g/100 g fresh sample. TFA is the sum of SFA, MUFA, and PUFA. SFA is the sum of C10:0, C12:0, C14:0, C15:0, C16:0, C17:0, C18:0, C21:0, and C24:0. MUFA is the sum of C14:1, C16:1, C17:1, C18:1n9t, and C18:1n9c. PUFA is the sum of C18:2n6t, C18:2n6c, C18:3n6, C18:3n3, C20:3n6, and C20:4n6.

The level of capric acid (C10:0) (*P* = 0.078) tended to be greater in the LT of lambs in the TRT group than in the CON group (Table 9). The LT of lambs in the TRT group had lower levels of myristoleic acid (C14:1) (*P* = 0.010) and heptadecenoic acid (C17:1) (*P* < 0.001). The levels of trans-linoleic acid (C18:2n6t) (*P* = 0.003) and gamma-linolenic acid (C18:3n6) (*P* < 0.001) were significantly lower in the TRT group than in the CON group. No significant differences were found between the two groups (Figure 3) for TFA (*P* = 0.32), SFA (*P* = 0.25), MUFA (*P* = 0.35) and PUFA (*P* = 0.59).

**Table 9.** Effects of replacing peanut vine by extruded rape straw on fatty acid profiles (g/100 g of fat) in the *longissimus thoracic* of lambs

Items	Groups		SEM	*P*-value
	CON	TRT		
C10:0	0.0037	0.0049	0.00033	0.078
C12:0	0.0025	0.0021	0.00037	0.619
C14:0	0.0469	0.0560	0.00351	0.201
C15:0	0.0068	0.0070	0.00083	0.315
C16:0	0.4920	0.5611	0.03165	0.287
C17:0	0.0208	0.0211	0.00205	0.436
C18:0	0.2942	0.3599	0.02488	0.195
C21:0	0.0017	0.0009	0.00077	0.311
C24:0	0.0028^a^	0^b^	0.00054	0.003
C14:1	0.0015^a^	0.0004^b^	0.00025	0.010
C16:1	0.0372	0.0421	0.00217	0.135
C17:1	0.0149^a^	0.0026^b^	0.00194	<0.001
C18:1n9t	0.0479	0.0517	0.00625	0.123
C18:1n9c	0.8583	0.9738	0.05223	0.280
C18:2n6t	0.0046^a^	0.0010^b^	0.00060	0.003
C18:2n6c	0.1666	0.1723	0.01251	0.280
C18:3n6	0.0020^a^	0^b^	0.00028	<0.001
C18:3n3	0.0051	0.0046	0.00056	0.622
C20:3n6	0.0053	0.0048	0.00052	0.850
C20:4n6	0.0968	0.1200	0.00768	0.105

^1^CON, peanut vine as the only roughage source; TRT, extruded rape straw as the only roughage source.

^a,b^Values in the same row with no common superscripts mean significant difference (*P* < 0.05).

## DISCUSSION

In this experiment, there was no difference in overall growth performance between lambs fed either peanut vine or extruded rape straw as the sole source of roughage. In a previous study, Zhang et al. (2016) observed a decrease in DMI (1.47 kg/d vs. 1.37 kg/d) in Hu sheep when the roughage proportion changed from 70% peanut vine to 58% rape straw. Similarly, in another study, lower DMI was found in Simmental crossbred steers fed 24.15 % wheat straw (9.58 kg/d) compared to those fed 24.15 % peanut vine (10.17 kg/d) (Zhu et al., 2022). These differences in DMI may be attributed to the significant difference in NDF content between peanut vine and rape straw (42.2 % vs. 54.3 %; Zhang et al., 2016) or rice straw (32.9 % vs. 40.5 %; Zhu et al., 2022), as NDF content directly influences the filling effect of the diet and thus DMI (Tedeschi et al., 2019). Nevertheless, we observed an interaction between dietary treatment and time, as ADG was greater in lambs in the TRT group from 60 to 90 d of age, indicating that the nutrients in extruded rape stalk-based diet could be more efficiently utilized during the 3^rd^ mo than the 2^nd^ mo of the experiment. Extrusion has been reported to reduce NDF content in diets or dietary ingredients (Solanas et al., 2005; Hejdysz et al., 2022), and our recent study confirmed that extrusion reduces NDF content in rape straw (Yang et al., 2024). Given the relatively low NDF content in the CON and TRT diets (27.9% vs. 30.7%), we expected no significant effects on the DMI of the two lamb groups. Of note, the FCR for Hu lambs averaged 4.90 kg DMI/kg BW gain throughout the trial period, which is greater than the range reported by Zhang et al. (2020; 4.15 to 4.82 depending on energy level), but lower than the 5.47 reported by Zeng et al. (2023). Another study with 653 Hu sheep found an FCR between 4.14 and 7.94 kg DMI/kg BW gain for growing lambs (Zhang et al., 2021). The differences in FCR among the studies can be attributed to differences in dietary composition, feeding strategies, and environmental conditions.

While the apparent digestibility of DM, NDF, and ADF did not differ significantly between the two groups of lambs, the apparent digestibility of EE was an exception. The lower proportion of dietary unsaturated FA in CON (0.541) vs TRT (0.701) may explain such difference as several studies showed that increasing unsaturation of fats increased apparent fatty acid digestibility in ruminants (Firkins and Eastridge, 1994; Harvatine and Allen, 2006). Beyond apparent digestibility, we investigated how different roughage sources affect energy metabolism in finishing lambs. As similar dietary GE content was measured between the CON (17.23 MJ/kg of DM) and TRT (16.96 MJ/kg of DM) groups, the greater GE intake in the TRT group was attributed to their greater DMI during the digestibility trial. Roughage source did not have an impact on overall energy utilization efficiency, as suggested by the insignificant digestibility (DE/GE) and metabolizability (ME/GE) of dietary energy between two groups of lambs. The ME intake was 0.803 and 0.965 MJ/(kg BW^0.75^ d) or 11.20 and 10.70 MJ/d for the lambs in the CON and TRT groups, respectively. These values are in agreement with the results of Wang et al. (2020), who found that an ME intake of at least 10.41 MJ/d is necessary to achieve an ADG of 250 g/d in growing female Hu lambs.

Total PD excretion varies between 0.518 and 0.697 mmol/ (kg BW^0.75^ d) and is influenced by concentrate-to-forage ratio and forage type (Carro et al., 2012). Previous studies have shown that allantoin is the predominant PD in sheep, accounting for about 70% to 80% of the total PD, followed by uric acid, xanthine and hypoxanthine (Ma et al., 2013; Zhou et al., 2017). In our study, allantoin accounted for more than 80% of total PD. In a recent study, allantoin levels ranging between 29.4% to 88.6% were found in various sheep studies (Del Valle et al., 2023). Sheep saliva contains considerable amounts of allantoin and contributes about 10% to urinary excretion through recycling (Chen et al., 1990). Although specific studies in sheep are limited, increasing the NDF content in the diet has been shown to prolong rumination time in dairy cows (Jiang et al., 2017; Shi et al., 2023). We thus speculated that the greater NDF intake of lambs in the TRT group during the digestibility trial might stimulate salivary excretion in sheep, resulting in a slightly greater amount of allantoin and a greater proportion of allantoin in total PD compared to sheep fed the CON diet. The marginally greater MCP synthesis of lambs in the TRT group was in accordance with the apparent digestibility of CP and the differences between dietary treatments in MCP synthesis. Again, a faster passage rate due to greater DMI in the TRT group may decrease nutrient degradability such as NDF in the rumen, however, undegraded NDF passing to the duodenum may serve an important vehicle to export microbial N flow considering that about 75% of the rumen bacteria were particle-associated (Sauvant and Nozière, 2016).

In our study, we identified 16 amino acids in the LT meat samples of Hu finishing lambs. However, the concentrations of Cys and Trp were not detected, which contrasts with previous results in lamb meat (Anaeto et al., 2010; Ding et al. 2024). Consistent with previous studies, Glu was the predominant amino acid in lamb (Belhaj et al., 2021; Ding et al. 2024), reflecting the high Glu content in the TRT diets used in this study. Glu and Asp contribute to the umami flavor of meat protein (Yamasaki and Maekawa 1978) and together accounted for up to 26.6% of TAA in this study. Apart from supplementation, branched-chain amino acids such as Val and Leu are primarily derived from MCP in the rumen and undegraded dietary protein (Zhang et al., 2017 ; Xu et al., 2022). Although the ruminal MCP calculated based on urinary PD excretion was slightly greater for lambs fed the TRT diet than those fed the CON diet, we hypothesize that the difference in undegraded dietary protein may compensate for this difference in the levels of Val and Leu, and finally resulting in insignificant levels of these branched-chain amino acids in LT meat from lambs fed extruded rape straw. In our experiment, the ratio of EAA to NEAA was above 0.65 in both groups, a value similar to that reported by Belhaj et al. (2021) and Jiao et al. (2023) for lamb, indicating good nutritional quality for human consumption.

The fatty acid profile is an important determinant of meat quality. In this study, similar levels of SFA, MUFA, PUFA, and TFA in the two groups of LT meat samples were observed, indicating that roughage source may not impact overall fatty acid profiles in LT meat. The TFA content was slightly greater than the values reported by Demirel et al. (2006), which is probably due to differences in breeds and feed. Palmitic acid (C16:0) and stearic acid (C18:0) contributed significantly to the SFA content, confirming the results of Jiao et al. (2023). Oleic acid (C18:1n9c) was the predominant MUFA, which is consistent with the observations of Vasilev et al. (2020) and is associated with improved meat flavor (Cameron et al., 2000) and positive effects on immune function and cholesterol regulation in humans (Sales-Campos et al., 2013). Previous studies have shown that the addition of rapeseed or rapeseed oil can reduce SFA and increase unsaturated fatty acids in lamb (Borys et al., 2005; Miltko et al., 2019). However, the extrusion process can change the fatty acid profiles in the feed, as some fatty acids may no longer be detectable in the feed after extrusion (Cian et al., 2017).

## CONCLUSIONS

In summary, replacing peanut vine with extruded rape straw in the diet did not have significant impact on growth performance, apparent digestibility of nutrients, MCP synthesis or meat quality of finishing lambs. These results suggest that extruded rape straw can effectively replace peanut vine in the diet of lambs without compromising health or nutrient utilization.
